# Application of group model building in implementation research: A systematic review of the public health and healthcare literature

**DOI:** 10.1371/journal.pone.0284765

**Published:** 2023-08-17

**Authors:** Weanne Myrrh Estrada-Magbanua, Terry T.-K. Huang, David W. Lounsbury, Priscila Zito, Pulwasha Iftikhar, Nabila El-Bassel, Louisa Gilbert, Elwin Wu, Bruce Y. Lee, Pedro Mateu-Gelabert, Nasim S. Sabounchi

**Affiliations:** 1 Center for Systems and Community Design and NYU-CUNY Prevention Research Center, CUNY Graduate School of Public Health and Health Policy, New York, NY, United States of America; 2 Division of Health Behavior Research and Implementation Science, Albert Einstein College of Medicine, New York, NY, United States of America; 3 Social Intervention Group, School of Social Work, Columbia University, New York, NY, United States of America; Aga Khan University Medical College Pakistan, PAKISTAN

## Abstract

**Background:**

Group model building is a process of engaging stakeholders in a participatory modeling process to elicit their perceptions of a problem and explore concepts regarding the origin, contributing factors, and potential solutions or interventions to a complex issue. Recently, it has emerged as a novel method for tackling complex, long-standing public health issues that traditional intervention models and frameworks cannot fully address. However, the extent to which group model building has resulted in the adoption of evidence-based practices, interventions, and policies for public health remains largely unstudied. The goal of this systematic review was to examine the public health and healthcare applications of GMB in the literature and outline how it has been used to foster implementation and dissemination of evidence-based interventions.

**Methods:**

We searched PubMed, Web of Science, and other databases through August 2022 for studies related to public health or health care where GMB was cited as a main methodology. We did not eliminate studies based on language, location, or date of publication. Three reviewers independently extracted data on GMB session characteristics, model attributes, and dissemination formats and content.

**Results:**

Seventy-two studies were included in the final review. Majority of GMB activities were in the fields of nutrition (n = 19, 26.4%), health care administration (n = 15, 20.8%), and environmental health (n = 12, 16.7%), and were conducted in the United States (n = 29, 40.3%) and Australia (n = 7, 9.7%). Twenty-three (31.9%) studies reported that GMB influenced implementation through policy change, intervention development, and community action plans; less than a third reported dissemination of the model outside journal publication. GMB was reported to have increased insight, facilitated consensus, and fostered communication among stakeholders.

**Conclusions:**

GMB is associated with tangible benefits to participants, including increased community engagement and development of systems solutions. Transdisciplinary stakeholder involvement and more rigorous evaluation and dissemination of GMB activities are recommended.

## Introduction

Group model building (GMB) is a process of engaging stakeholders in a participatory modeling process to elicit their perceptions of a problem and explore concepts regarding the origin, contributing factors, and potential solutions or interventions to a complex issue [1, 2]. For decades, GMB had been widely used in fields such as business, public policy, criminal justice, and environmental resource planning, among others [3]. More recently, however, it has emerged as a novel method for tackling complex, long-standing public health issues that traditional intervention models and frameworks cannot fully address.

In the past, conventional public health approaches have tended to lean towards more downstream, compartmentalized interventions that do not take into account the whole-system perspective. However, these approaches have been insufficient, as many public health issues meet the key criteria defining a dynamic complex system as defined by Sterman [4]: (a) strong interactions between the various actors of the system, (b) constant fluctuations and changes in trends and behaviors over time, (c) an internal complex causal structure subject to feedbacks from the different actors within the system, and (d) long delays between actions and effects. When not considered, these characteristics can lead to counterintuitive, unintended consequences that may be difficult to predict. For example, past attempts to address syndemic issues such as substance abuse through well-intentioned but single-perspective policies (e.g. increased policing or increased dispensing of medications for opioid use disorders) have instead, in the long term, led to a reinforcing cycle of stigma and compassion burnout [5, 6]. Therefore, it is evident that addressing these dynamically complex public health problems more effectively requires a shift from the traditional linear ways of thinking to a more holistic manner of examining the context, relationships, and systems within which they exist. GMB offers an organized, collaborative approach towards achieving that goal.

**Fig 1:**
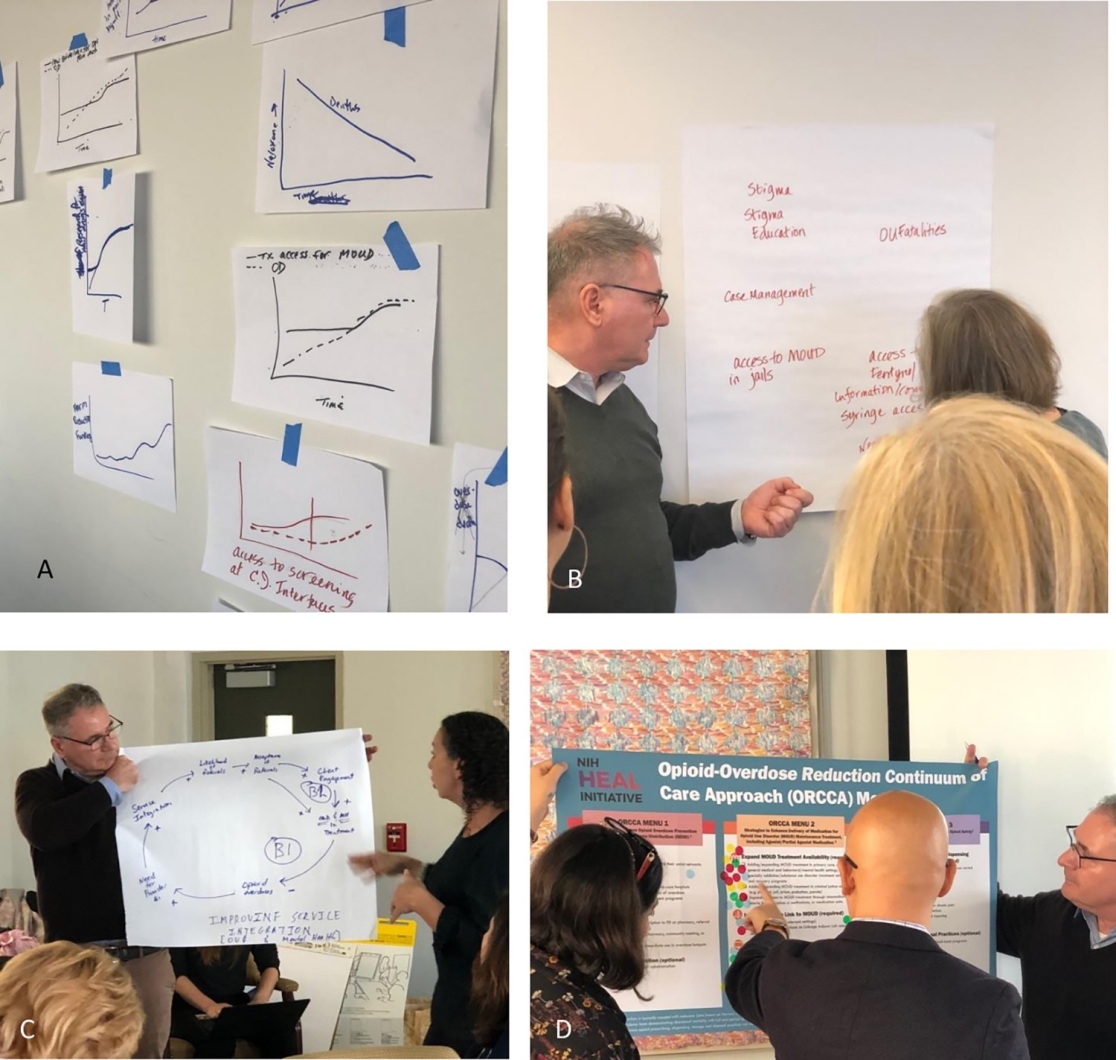
Images of GMB activities. (A) Graphs over time: graphs drawn by participants depicting the behavior of variables over time. (B) Variable elicitation: variables affecting opioid use elicited from participants. (C) Creating a CLD: a CLD indicating connections and feedback behaviors between variables developed by participants (D) Dots exercise to address priorities: Output of a dots exercise demonstrating priority actions ranked by participants.

GMB adopts system dynamics (SD) methodology, which is a set of principles for framing and understanding the nonlinear behavior of complex systems as they change over time using feedback loops and stocks and flows [7]. SD involves a sequence of five stages: articulation of the problem that needs to be solved, formulation of a hypothesis that conceptualizes the dynamics of the system, formulation of a simulation model representing the system, model testing, and policy design and evaluation. In GMB, a purposefully selected, ideally diverse range of stakeholders, such as policy makers, community leaders, and persons with lived experience who can inform the project’s focus are engaged in these stages through a sequence of scripted group exercises carried out under the guidance of a skilled modeling team [8]. Scripts are used to direct facilitated structured small group exercises that ultimately inform the design, development and application of a systems dynamics model. Depending on the purpose of the GMB, facilitators select and carry out two or more scripts typically built around the following order of tasks (a) presentation (i.e. orienting or explaining SD concepts to participants) (b) divergent thinking (i.e. brainstorming), (c) convergent thinking (i.e. building consensus, shared understanding, or shared decision-making), and (d) evaluation (i.e. group assessment/group ranking of ideas). Table 1 presents examples of classic GMB scripts for each task. The end products of these activities are presented as causal loop diagrams (CLD) and/or stock and flow diagrams which can be simulated to explore the problem of interest (Fig 1) [9].

**Table 1:** Examples of GMB scripts.

Script^a^	Description
A Graphs Over Time	Participants are asked to draw multiple graphs of variables over time showing important characteristics of the issue at hand. For example, if the issue is opioid use disorder, the graph over time could be used to sketch rates of opioid use, overdose deaths, access to preventive or treatment services in the community, over time.
B Variable Elicitation	The facilitators ask participants, “what are the key variables affecting the process and outcomes of the problem at hand?” Participants write down as many problem-related variables as they can on sheets of paper and then share their list with the rest of the group. The facilitators tape the variables to a wall and cluster them according to common themes. The facilitators then prompt discussion by asking participants questions such as, “Does this resonate with you? Are there other themes you notice, or any variables you think should be moved?”
C Creating a Causal Loop Diagram	The facilitators present the variables elicited from the group in the previous activity and ask the participants how the variables from the list interact and cause changes to the problem at hand. The group indicates the relationship between the variables by linking them and labeling the link with a positive (i.e. an increase in one variable will cause an increase in the other variable) or negative (i.e. an increase in one variable will cause a decrease in the other variable) polarity.
D Dots	This exercise is used when there is a need to select variables, graphs, or ideas that are most important to the participant group. For example, after the variable elicitation script, participants vote on what they perceive to be the most significant variables affecting the problem at hand by placing voting dots beside the elicited variables. The facilitators then tally the dots beside each item to create a ranked list of importance.

^a^ A full list of scripts may be accessed on Scriptapedia, a publicly available online repository of GMB scripts [10].

As the GMB process builds on an iterative cycle of collective deliberation, stakeholder consultation and verification, and collaborative modeling, the resultant models integrate scientific knowledge and practical experience [11], leading to the identification of systems strategies for intervention that addresses the target problem [12, 13]. Moreover, the added value of stakeholder engagement fosters group ownership of the model and engenders a shared insight of the system behavior surrounding the problem. Overall, the success of GMB lies not just in the construction of the model itself but in the process it takes to get there [14].

Applying GMB as a method to optimize processes for implementing interventions is fairly recent in public health and healthcare. Nonetheless, studies that document its application in these fields have begun to emerge in the past decade, providing insight into the extent to which it may be applicable across health issues. What remains largely unstudied is the extent to which GMB has resulted in the adoption and integration of evidence-based practices, interventions, and policies for public health. Further, despite the increase of GMB in social and behavioral research, no systematic review of its application and effectiveness in public health and healthcare has yet been conducted.

This paper is designed to examine the literature on GMB and how it has been used and disseminated to foster implementation of evidence-based interventions (EBIs) in public health and healthcare. The following research questions frame our systematic review: (a) where is the application of GMB most prevalent in public health and healthcare; (b) how have GMB activities and strategies been disseminated and applied to improve EBI implementation and systems thinking capacities; and (c) what individual, group/organizational, or community level outcomes have resulted from GMB activities.

## Methods

### Search strategy

**Fig 2:**
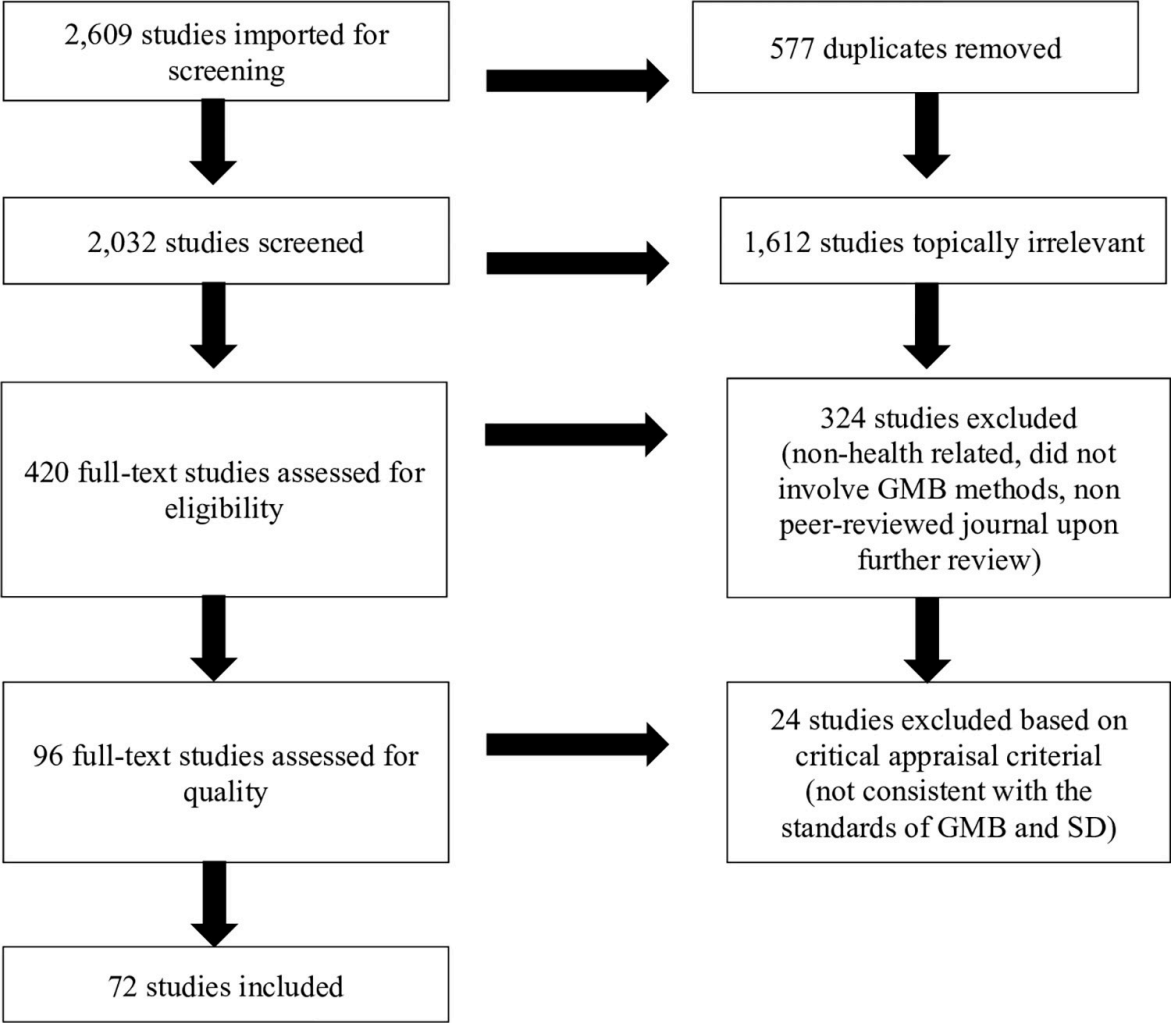
PRISMA diagram.

This is a systematic review of GMB applications in public health and implementation science. The Preferred Reporting Items for Systematic Reviews and Meta-Analyses (PRISMA) Guidelines were used to delineate each step of the review process (Fig 2).

We consulted seven electronic databases (CINAHL, Embase, Web of Science, PubMed, ProQuest, Scopus and EBSCO) to ensure an exhaustive coverage of the literature from any year up to August 2022 using the following keywords: *("system dynamics modeling" OR "group model building" OR "participatory system dynamics" OR "participatory model building" OR "community based system dynamics" OR "mediated modeling" OR "collaborative modeling" OR "heterogenous problem solving" OR "participatory stakeholder engagement" OR "collaborative model building”) AND (“implementation” OR “dissemination”)*. After studies were selected through our inclusion criteria for the final review, the reference list of selected studies and systematic reviews were also checked as a secondary method of identifying relevant publications.

### Inclusion and exclusion criteria

We included studies where research questions were related to public health or health care and GMB was cited as one of the main methodologies. We did not eliminate studies from our search based on language, geographic location, or date of publication. Systematic reviews, theoretical papers, and articles missing explicit mentions of GMB or its related terms were excluded from this study.

We used Covidence, an online systematic review software, to conduct study screening. Three authors (WE, PZ, and PI) independently screened all titles, abstracts, and full-text articles and noted reasons for excluding studies during full-text review. Differences in screening decisions were resolved by the corresponding author (NS) who served as a third independent reviewer. The decisions were also supported by a group discussion on the article’s eligibility until consensus was reached.

After screening, three authors (WE, NS, DL) conducted a critical appraisal on the final articles to ensure the quality of studies included in this systematic review. Studies were considered good quality if they met a minimum set of criteria defined by the study authors as consistent with the standards of GMB and SD, namely, (a) the study explicitly carried out activities or scripts that support collaborative model building, and (b) the study correctly described SD concepts (e.g. polarity of the causal links are indicated, the type of feedback loops are discussed, stocks and flows are well specified, and/or resulting simulation results are presented) in the results or discussion sections.

### Data extraction

Three authors (WE, PZ, and PI) independently piloted a structured abstraction form on ten studies; the final abstraction form was approved by three co-authors (TH, DL, and NS). One of two authors (PZ and PI) independently extracted data such as participant type, GMB team composition, GMB session characteristics, model attributes, model dissemination, implementation effects, and outcomes from the included studies onto a shared spreadsheet. A third author (WE) reviewed each of the abstractions against the original articles to ensure accuracy and consistency.

The models resulting from the GMB sessions were coded by the reviewers into three developmental phases or stages of validation. Studies were considered to be in the d*emonstration phase*, the first phase, if they involved developing a high-generality, low-resolution scoping and consensus-building model generated by the stakeholder groups involved in GMB. They were considered to be in the *research modeling* phase if they were characterized by the use of historical data to calibrate and test the model developed during the demonstration phase. Finally, they were in the *management* phase if they involved running different scenarios across the model to identify the most effective intervention and policy options [15].

Each of the GMB activities mentioned in the studies were coded as a GMB script based on the definitions provided on the free Scriptapedia website [10]. For example, GMB activities mentioned in the studies that asked participants to write down a list of key variables affecting a phenomenon or public health issue were coded as “Variable Elicitation,” as they corresponded with the definition for that script on Scriptapedia.

The studies were also reviewed to identify whether or not the outcomes proposed by Rouwette et al. [16] occurred as a result of the GMB sessions. These outcomes include *reaction*, *insight*, *commitment*, *behavior*, *communication*, *consensus*, *shared language*, *system changes*, *results*, *further use method*, and *efficiency* and are classified under four levels (*individual*, *group*, *organization*, and *project*). Three new variables, namely *systems thinking skills*, *confidence in the model*, and *improved understanding of role and responsibility* were also added as outcomes to reflect common GMB goals and findings reported in the literature [17–19]. These variables were coded positively if a study reported outcomes that were consistent with the definitions provided in Table 2. Outcomes that were not found or reported in the articles were coded as “*not mentioned”*.

**Table 2:** GMB outcomes adapted from Rouwette et al. [16].

Level	Outcome	Definition
**Individual**	New insights	Gaining new perspectives or a deeper understanding about systems problems
	Positive reaction	Positive personal evaluation of GMB or the resultant model
	Systems thinking skills^a^	Development or improvement in ability to think about time-dependent patterns of change and causality
	Commitment	Decisive commitment to results attributable to GMB experience
	Improved understanding of role or responsibility^a^	Improved understanding of individual role or responsibility within the system
	Behavior	Changes in personal strategies or approaches attributed to GMB experience
**Group**	Consensus	Building a shared understanding of the problem and possible solutions
	Communication	Change in the quality of communication among participants
	Confidence in model^a^	Trust in the model and its performance (i.e., structural and behavioral validity)
	Shared language	Understanding of other participants
**Organization**	System changes	Organizational or physical changes (e.g., production lines, personnel policies)
	Positive results of system changes	Desirable outcomes associated with model-informed policies and actions (e.g., increased profits or improved morale)
**Project**	Further use of SD methods	Intention to use SD methods for future projects
	Efficiency	Efficiency of GMB compared to traditional methods (e.g., a meeting)

^a^Additional codes identified by study authors

We calculated percentages for categorical variables and means and medians for continuous variables. We did not conduct a meta-analysis of the study findings due to the heterogeneity in how the outcomes were reported and presented across all the articles.

## Results

The search yielded 2,032 unique articles for title and abstract screening, of which 420 articles were deemed potentially relevant and selected for full text review. A total of 324 studies was then excluded as further in-depth review revealed that they were not related to public health or health care, did not involve GMB methods, or were not published in a peer-reviewed journal. The critical appraisal of the remaining studies led to the additional exclusion of 24 studies as they did not meet the minimum criteria for quality. In these studies, modeling activities were not explicitly described or conducted, or the feedback loops, polarities, or stock and flow diagrams developed were not consistent with the standards of SD. In the end, 72 studies published from 2002 to 2022 were included in the final review. Fig 2 shows the selection of studies for review based on PRISMA while Table 3 outlines the list of all included studies and their outcomes.

**Table 3:** List of studies in the systematic review.

Study	Project Purpose	Public Health Domain	Model Level of Analysis	Developmental Stage/Stage of Validation	Mode of Dissemination	Did GMB inform intervention design?	Outcomes
Calancie et al., 2022 (United States) [20]	Build a valid model Build rapport across and among stakeholders Design/improve implementation	Nutrition	Community/ population	Demonstration	Open source White paper	Yes	Positive reaction Insight Communication System change Improved understanding of role or responsibility Positive results of system changes
Gullett et al., 2022 (United States) [21]	Build a valid model Design/improve implementation	Social work and development	Community/ population	Management	Open source Software interface	Not mentioned	Insight Behavior change Communication Consensus Shared language Systems thinking skills Further use of SD methods
Hendricks et al., 2022 (South Africa) [22]	Build a valid model	Nutrition	Individual	Demonstration	Open source	Not mentioned	Insight
Broekhuizen et al., 2021 (Zambia) [23]	Design/improve implementation	Health care/Hospital administration and management	Group/ organization	Demonstration	Open source Online file sharing	Not mentioned	Insight Communication Consensus
Broekhuizen et al., 2021 (Malawi) [24]	Build a valid model Foster decision making Design/improve implementation	Health care/Hospital administration and management	Group/ organization	Management	Open source	Not mentioned	Insight
Susnik et al., 2021 (Latvia) [25]	Build a valid model Design/improve implementation	Environmental health	Community/ population	Management	Non-open source	Not mentioned	Insight
Shire et al., 2020 (United Kingdom) [26]	Build a valid model Foster decision mking Design/improve implementation	Health care/Hospital administration and management Pharmaceuticals	Group/ organization	Management	Non-open source Software interface	Not mentioned	Positive reaction Insight Confidence in model Communication Consensus Shared language Improved understanding of role or responsibility Systems thinking skills Further use of SD methods Efficiency
Guarigata et al., 2020 (Jamaica, St. Kitts and Nevis, St. Vincent, the Grenadines) [27]	Build a valid model Design/improve implementation	Nutrition	Community/ population	Demonstration	Open source	Not mentioned	Positive reaction Insight Commitment Confidence in model Communication Consensus Systems thinking skills
Sarmiento et al., 2020 (Colombia) [28]	Build a valid model Build rapport across and among stakeholders Foster decision making	Infrastructure	Community/ population	Demonstration	Open source	Yes	Insight
Weeks et al., 2020; Weeks et al., 2017 (United States) [29, 30]	Build a valid model Foster decision-making Design/improve implementation	Infectious diseases	Community/ population	Research	Open source Software interface Planning meeting	Not mentioned	Positive reaction Insight Commitment Confidence in model Communication Consensus Shared language Systems thinking skills
Hennessy et al., 2020; Appel et al., 2019 (United States) [31, 32]	Build a valid model Design/improve implementation	Non-communicable diseases Nutrition	Community/ population	Demonstration	Non-open source	Yes	Insight Confidence in model Communication Shared language Further use of SD methods
Naumann et al., 2020 (United States) [33]	Build a valid model	Infrastructure	Community/ population	Demonstration	Non-open source	Not mentioned	Insight Communication Systems thinking skills
McGlashan et al., 2018 Allender et al., 2015; (Australia) [34, 35]	Build a valid model	Nutrition	Community/ population	Demonstration	Open source Community meeting	Yes	Positive reaction Insight Commitment Behavior Communication Consensus Shared language System changes Systems thinking skills Improved understanding of role or responsibility Positive results of system changes Further use of SD methods Efficiency
Langellier et al., 2019 (Peru, Brazil, Guatemala) [36]	Build a valid model Build rapport across and among stakeholders	Nutrition Infrastructure	Community/ population	Demonstration	Open source Written report	Yes	Positive reaction Insight Communication Consensus Shared language Systems thinking skills Further use of SD methods
Escobedo et al., 2019 (United States) [37]	Build a valid model Design/improve implementation	Mental health/addiction and substance abuse	Community/ population	Demonstration	Non-open source	Not mentioned	Insight Consensus System changes Improved understanding of role or responsibility
Fowler et al., 2019 (United States) [38]	Build a valid model Build rapport across and among stakeholders	Social work and development	Group/ organization	Demonstration	Non-open source Written report Organizational presentations	Not mentioned	Insight Confidence in model Communication Consensus Improved understanding of role or responsibility
Dianati et al., 2019 (Kenya) [39]	Build a valid model Foster decision making	Environmental health	Community/ population	Management	Open source	Not mentioned	Positive reaction Insight Confidence in model Consensus Systems thinking skills
Ansah et al., 2019 (Cambodia) [40]	Build a valid model Build rapport across and among stakeholders	Non-communicable diseases	Community/ population	Demonstration	Open source	Not mentioned	Positive reaction Insight Communication Consensus Further use of SD methods
Gamble et al., 2019 (United States) [41]	Build a valid model Build rapport across and among stakeholders Design/improve implementation	Health care/Hospital administration and management	Group/ organization	Demonstration	Non-open source	Yes	Positive reaction Insight Commitment Behavior Communication Shared language Systems change Systems thinking skills Positive results of system changes Further use of SD methods
Qayoom et al., 2019 (Pakistan) [42]	Build a valid model	Occupational health	Group/ organization	Management	Non-open source	Not mentioned	Insight Confidence in model
Hosseinichimeh et al., 2019 (United States) [43]	Build a valid model	Maternal and child health	Community/ population Group/ organization Individual	Management	Non-open source Website Organizational presentation	Not mentioned	Insight Communication Shared language Improved understanding of role or responsibility Further use of SD methods Efficiency
Baker et al., 2019 (Switzerland) [44]	Build a valid model Design/improve implementation Foster decision making	Nutrition, Health care/Hospital administration and management	Group/ organization	Demonstration	Non-open source	Not mentioned	Insight
Gerritsen et al., 2019 (New Zealand) [45]	Build a valid model Design/improve implementation	Nutrition	Community/ population	Demonstration	Open source	Yes	Positive reaction Insight Commitment System changes Further use of SD methods
Urwannachotima et al., 2019 (Thailand) [46]	Build a valid model Foster decision-making	Nutrition	Community/ population	Demonstration	Open source	Not mentioned	Insight Confidence in model Communication Consensus Further use of SD methods
Purwanto et al., 2019 (Indonesia) [47]	Build a valid model Build rapport across and among stakeholders Foster decision making	Environmental health	Community/ population	Demonstration	Non-open source	Not mentioned	Positive reaction Insight Consensus Improved understanding of role or responsibility Systems thinking skills
Roberts et al., 2019 (Australia) [48]	Build a valid model Foster decision making	Nutrition	Community/ population	Management	Open source	Not mentioned	Insight Communication Consensus Further use of SD methods Efficiency
Mui et al., 2019 (United States) [49]	Build a valid model Build rapport across and among stakeholders Design/improve implementation	Nutrition	Community/ population	Demonstration	Open source	Not mentioned	Insight Communication Further use of SD methods Efficiency
Pagano et al., 2019 (Slovenia) [50]	Build rapport across and among stakeholders Design/improve implementation	Environmental health	Community/ population	Management	Open source Stella interface	Yes	Positive reaction Insight Communication Consensus Shared language Further use of SD methods
Malard et al., 2018 (Mexico) [51]	Build a valid model Foster decision-making	Environmental health	Community/ population	Demonstration	Open source Conference presentation	Not mentioned	Positive reaction Insight Communication Consensus Improved understanding of role or responsibility Further use of SD methods
Koh et al., 2018 (United States) [52]	Build a valid model	Nutrition	Community/ population	Research	Open source Website Online file sharing program	Not mentioned	Insight Confidence in model Shared language Systems thinking skills Efficiency
Ansah et al., 2018 (Singapore) [53]	Build a valid model	Health care/Hospital administration and management	Group/ organization	Demonstration	Non-open source	Not mentioned	Insight Systems thinking skills Further use of SD methods
Eker et al., 2018 (United Kingdom) [54]	Build a valid model Foster decision making	Environmental health Infrastructure	Community/ population	Research	Open source Stakeholder presentation Written report	Not mentioned	Positive reaction Insight Confidence in model Communication Consensus Systems thinking skills Further use of SD methods Efficiency
Chalise, 2018 (India) [55]	Build a valid model	Environmental health	Community/ population	Management	Open source	Not mentioned	Insight Confidence in model Communication
Lembani et al., 2018 (South Africa) [56]	Build a valid model Build rapport across and among stakeholders	Maternal and child health	Group/ organization	Demonstration	Open source Written report Stakeholder presentation	Yes	Insight Commitment Consensus Shared language System changes Improved understanding of role or responsibility Efficiency
Reno, 2018 (United States) [57]	Build a valid model	Maternal and child health	Community/ population	Demonstration	Non-open source	Not mentioned	Insight Communication Consensus
Brown et al., 2018 (Australia) [58]	Build a valid model Foster decision making	Environmental health	Community/ population	Demonstration	Open source	Not mentioned	Insight
Atkinson et al., 2017 (Australia) [59]	Build a valid model Build rapport across and among stakeholders Foster decision making Design/improve implementation	Mental health/addiction and substance abuse	Community/ population	Management	Open source	Not mentioned	Insight Consensus Further use of SD methods
Freebairn et al., 2017 (Australia) [60]	Build a valid model Build rapport across and among stakeholders Design/improve implementation	Mental health/addiction and substance abuse	Community/ population	Management	Open source	Yes	Insight Confidence in model Communication Consensus Shared language Systems thinking skills
Kopainsky et al., 2017 (Zambia) [61]	Build a valid model Foster decision-making	Nutrition	Individual	Demonstration	Non-open source Written report	Yes	Positive reaction Insight Behavior Communication Consensus Improved understanding of role or responsibility Systems thinking skills
Morrow-Howell et al., 2017 (United States) [62]	Design/improve implementation	Social work and development	Community/ population	Demonstration	Open source	Not mentioned	Insight Further use of SD methods Efficiency
Macmillan et al., 2017 (United Kingdom, the Netherlands) [63]	Build a valid model Design/improve implementation	Infrastructure	Community/ population	Demonstration	Open source Written report	Not mentioned	Insight Communication
Mumba et al., 2017 (Zambia) [64]	Design/improve implementation Foster decision making	Animal health	Community/ population	Demonstration	Open source	Not mentioned	Positive reaction Insight Consensus Systems thinking skills
Waqa et al., 2017 (Fiji) [20]	Build a valid model Design/improve implementation	Nutrition	Community/ population	Demonstration	Open source	Yes	Insight Commitment Consensus System changes Improved understanding of role or responsibility Positive results of system changes
Jetha et al., 2017 (United States) [65]	Build a valid model	Mental health/addiction and substance abuse	Group/ organization	Management	Open source	Not mentioned	Insight Consensus Improved understanding of role or responsibility Efficiency
Trani et al., 2016 (Afghanistan) [66]	Foster decision making Design/improve implementation	Mental health/addiction and substance abuse	Community/ population	Demonstration	Open source	Not mentioned	Positive reaction Insight Commitment Communication Consensus Shared language Improved understanding of role or responsibility Efficiency
Jetha et al., 2016 (United States) [67]	Build a valid model	Occupational health	Group/ organization	Management	Open source	Insight	
Frerichs et al., 2016 (United States) [68]	Build a valid model	Crime/violence	Community/ population	Demonstration	Open source Stakeholder presentation	Not mentioned	Positive reaction Insight Commitment Confidence in model Communication Systems thinking skills Further use of SD methods
Zimmerman et al., 2016 (United States) [69]	Build a valid model Design/improve implementation	Health care/Hospital administration and management	Group/ organization	Management	Non-open source	Yes	Positive reaction Insight Consensus Shared language System change Systems thinking skills Positive results of system changes Further use of SD methods Efficiency
Macmillan et al., 2016 (United Kingdom) [70]	Build a valid model Foster decision making Design/improve implementation	Environmental health	Community/ population	Demonstration	Open source Website File copy	Not mentioned	Insight Commitment Consensus Systems thinking skills
Brownson et al., 2015 (United States) [71]	Build rapport across and among stakeholders	Health care/Hospital administration and management	Group/ organization, Community/ population	Demonstration	Open source	Yes	Positive reaction Insight Commitment Confidence in model Behavior change Communication Shared language System changes Improved understanding of role or responsibility Systems thinking skills Positive results of system changes Efficiency
Homa, 2015 (United States) [72]	Build a valid model Foster decision-making	Health care/Hospital administration and management	Community/ population	Research	Open source Website, blog, or social media post	Not mentioned	Insight Efficiency
Thomas et al., 2015 (United States) [73]	Foster decision-making Design/improve implementation	Nutrition	Community/ population	Demonstration	Non-open source	Yes	Positive reaction Insight Commitment Confidence in model Communication Consensus Shared language Systems change Efficiency
Brennan et al., 2015 (United States) [74]	Foster decision-making Design/improve implementation	Nutrition	Community/ population	Demonstration	Non-open source	Not mentioned	Insight Commitment Communication Consensus Shared language Improved understanding of role or responsibility Efficiency
Munar et al., 2015 (Honduras) [75]	Foster decision-making	Maternal and child health	Community/ population	Research	Non-open source	Yes	Insight Systems thinking skills Further use of SD methods
Keane et al., 2015 (United States) [76]	Build a valid model Design/improve implementation	Nutrition	Community/ population	Demonstration	Non-open source	Yes	Insight Commitment Confidence in model Behavior change Communication Shared language Systems change Positive results of system change Further use of SD methods
Moreland, 2015 (United States) [77]	Build a valid model	Nutrition	Community/ population	Demonstration	Non-open source	Yes	Insight Behavior change Systems thinking skills Efficiency
Skouteris et al., 2015 (Australia) [78]	Build a valid model Design/improve implementation	Nutrition	Community/ population	Demonstration	Non-open source	Yes	Insight Consensus
Esensoy et al., 2015 (Canada) [79]	Build a valid model Foster decision making Design/improve implementation	Health care/Hospital administration and management	Group/ organization Community/ population	Demonstration	Open source Panel presentations	Not mentioned	Positive reaction Insight Commitment Confidence in model Communication Consensus Shared language Systems change Improved understanding of role or responsibility Positive results of systems change Efficiency
Ager et al., 2015 (Nigeria) [80]	Build a valid model	Health care/Hospital administration and management	Group/ organization	Demonstration	Open source Stakeholder presentation	Not mentioned	Insight
Van Nistelrooij et al., 2015 (Netherlands) [81]	Build a valid model	Health care/Hospital administration and management	Community/ population	Research	Non-open source	Not mentioned	Insight Confidence in model Consensus Further use of SD methods
Macmillan et al., 2014 (New Zealand) [82]	Foster decision making Design/improve implementation	Infrastructure	Community/ population	Management	Open source	Not mentioned	Insight Communication Consensus
Narayana et al., 2014 (India) [83]	Build a valid model	Pharmaceuticals	Community/ population	Demonstration	Non-open source Written report	Not mentioned	Insight Consensus Further use of SD methods
Biroscak et al., 2014 (United States) [84]	Build a valid model	Health care/Hospital administration and management	Group/ organization	Demonstration	Non-open source	Not mentioned	Insight
Rouwette et al., 2014 (Netherlands) [85]	Build a valid model Build rapport across and among stakeholders	Crime/violence	Community/ population	Demonstration	Open source Written report	Yes	Positive reaction Insight Confidence in model Communication Consensus Shared language System change Improved understanding of role or responsibility Positive results of system changes
Merrill et al., 2013 (United States) [86]	Build a valid model Foster decision-making Design/improve implementation	Health care/hospital administration and management	Group/ organization	Demonstration	Open source	Yes	Insight Confidence in model Consensus Efficiency
Hernantes et al., 2012 (Spain) [87]	Build a valid model Foster decision making	Crisis and emergencies Infrastructure	Community/ population	Management	Open source Website	Not mentioned	Positive reaction Insight Confidence in model Communication Consensus Systems thinking skills
Goh et al., 2012 (Australia) [88]	Build a valid model	Occupational health	Group/ organization	Demonstration	Non-open source	Yes	Insight Commitment Confidence in model Systems thinking skills Further use of SD methods Efficiency
Bridgewater et al., 2010 (United States) [89]	Build rapport across and among stakeholders Foster decision-making Design/improve implementation	Crime/violence	Community/ population	Research	Open source	Not mentioned	Positive reaction Insight Communication Consensus Shared language Systems thinking skills Efficiency
Thompson et al., 2010 (United States) [90]	Build a valid model Foster decision making	Environmental health	Community/ population	Research	Non-open source File copy	Not mentioned	Positive reaction Insight Commitmet Confidence in model Behavior change Communication Improved understanding of role or responsibility Systems thinking skills
Stave, 2010 (United States) [91]	Build a valid model Design/improve implementation	Environmental health	Community/ population	Management	Open source Written report Conference presentation Oral presentation File copy	Not mentioned	Positive reaction Insight Commitment Confidence in model Behavior change Communication Consensus Shared language Systems thinking skills Further use of SD methods
Cavana et al., 2006 (New Zealand) [92]	Build a valid model	Health care/Hospital administration and management	Community/ population	Management modeling	Non-open source Conference presentation	Not mentioned	Positive reaction Insight Confidence in model Behavior change Consensus Shared language System change Improved understanding of role or responsibility Further use of SD methods Efficiency
Stave, 2002 (United States) [93]	Build a valid model Build rapport across and among stakeholders Foster decision-making Design/improve implementation	Environmental health	Community/ population	Management	Non-open source Stakeholder presentation	Yes	Positive reaction Insight Behavior change Communication Consensus Shared language Systems thinking skills Efficiency

### Study information

More than half of the documented studies on public health applications of GMB were published in the last five years from 2017–2022 (n = 42, 58.3%). Most of the studies were conducted in high-income countries, particularly the United States (n = 29, 40.3%) and Australia (n = 7, 9.7%), although studies were found across six continents and across the different country-level income classifications as defined by the World Bank [94]. GMB methods were most heavily utilized in the domains of nutrition (n = 19, 26.4%), health care/hospital administration (n = 15, 20.8%), and environmental health (n = 12, 16.7%). GMB was conducted for the following purposes: to build a valid model (n = 59, 81.9%), to design/improve implementation (n = 34, 47.2%), to foster decision-making (n = 29, 40.3%), and to build rapport among stakeholders (n = 16, 22.2%).

### Session characteristics

The characteristics of the GMB sessions varied widely across studies. The number of participants in the sessions ranged from as few as five people to as many as 50 people in one session, with 15 as the median number of participants. Most of the studies required two to three sessions; however, some only had one session while one study required 22. Each session lasted about 1.5 hours to a full day, with a mean duration of four hours.

GMB relies on participants who represent relevant, diverse stakeholder groups and experts [9]. Among all the participant types in this review, community coalition members/advocacy groups (n = 39, 54.2%) were the most often engaged in GMB projects, followed by government officials (n = 38, 52.8%) and domain experts (n = 34, 47.2%). The correctional or justice system (n = 4, 5.6%) and funding agencies (n = 2, 2.8%) were the least commonly represented.

GMB sessions were typically directed by a school- or university-based team with training in SD modeling. Actual facilitation of the sessions was conducted by a GMB team of, on average, seven persons who took on roles such as conveners, community facilitators, modelers, recorders, note-takers, and observers [8]. The most common GMB scripts mentioned in the literature were causal loop diagramming (n = 46, 63.9%; convergent thinking), variable elicitation (n = 39, 54.2%; divergent thinking), concept models (n = 29, 40.3%; presentation), graphs over time (n = 21, 29.2%; divergent thinking), and action ideas (n = 21, 29.2%; divergent thinking, evaluation).

Process evaluations determine whether workshops are implemented as intended and resulted in meaningful outputs. Only 11 of the 72 studies (15.3%) reported conducting a process evaluation to assess the implementation of the workshops and their fidelity to stated objectives of the GMB sessions.

### Model attributes

The majority of the modelers and system dynamicists reported utilizing Vensim (Ventana Systems, Harvard, MA) (n = 36, 50.0%) and Stella Architect (ISEE Systems) (n = 7, 9.7%) to create and refine the models that emerged from the GMB workshops. The models mostly included qualitative (CLDs and stock-and-flow figures) in nature (n = 45, 62.5%), but 26 studies (36.1%) involved quantitative or simulation modeling. Most of the models and simulations modeled the overall dynamics of a community (n = 54, 75.0%), 19 (26.4%) modeled the dynamics of a group or organization, while three (4.2%) centered only on individual factors and behaviors. Majority of the models created by stakeholder groups reached only the demonstration phase of modeling (n = 44, 61.1%), while 18 (25.0%) studies reached the management modeling phase.

### GMB outcomes

Twenty-three (31.9%) studies reported that the findings and models from the GMB workshops influenced implementation through policy change, intervention development, and/or community action plans. These models were used to develop local action plans, revise intervention approaches in grant applications, guide strategic planning and agendas, publish guidelines and communication messages, develop new partnerships, and activate policy leverage points. The remaining studies (n = 49, 68.1%) did not report whether GMB influenced or contributed to implementation.

**Fig 3:**
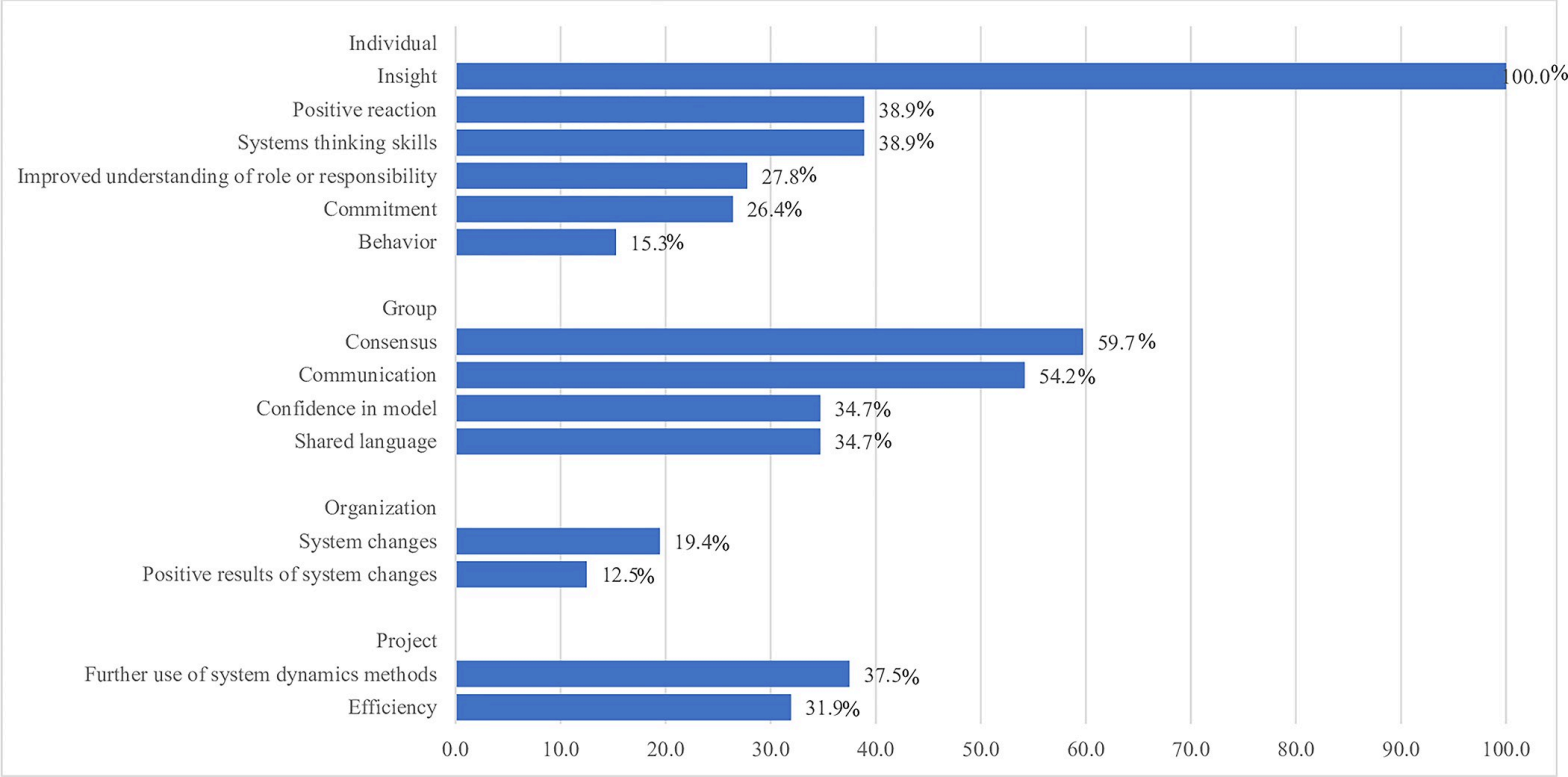
Outcomes identified in the GMB studies.

*Insight*, *consensus*, and *communication* were the most commonly reported outcomes of the papers reviewed (Fig 3). Insight is defined by Rouwette et al. as individual-level learning [16]. We documented that all studies (n = 72, 100%) in our review generated useful insights among participants. In studies that apply GMB, greater insights among participants are produced if the problem to be modeled aligns with their interests and priorities, and if modeling efforts are sufficient to support the aims of the study [16].

Consensus or mental model alignment is a group-level outcome defined as a shared view of the problem or actions among GMB participants. It is considered a prerequisite for shared action [16]. Forty-three (59.7%) of the articles in this review indicated that consensus was achieved after the workshops. A common strategy mentioned in the literature to achieve consensus was the use of CLDs as boundary objects [36]. Boundary objects are representations, such as a diagram, sketch, or prototype, that help individuals collaborate effectively across a boundary such as a difference in knowledge, training, or objective [95]. Other strategies to build consensus that were mentioned in the literature were voting [60, 64] and continued discussion until all concerns were addressed [86].

A change in the quality of communication among GMB participants is necessary for consensus to emerge [16]. Thirty-nine (54.2%) of the articles in this review explicitly mentioned that an improvement in communication among stakeholders occurred during the GMB workshops. Participatory methods such as GMB generally imply the presence of communication; it is possible that this outcome actually occurred more than was reported and was just not explicitly documented as it was thought to be obvious.

### Model dissemination

Model dissemination was high, with 70 (97.2%) of the studies publishing the CLDs or the full models developed through GMB. However, while the workshop activities and resultant were disseminated actively to the academic community through peer-reviewed publications in open-source journals (n = 43, 59.7%), information on whether the models were shared back to stakeholders or the non-academic community was limited. Studies that reported doing so (n = 20, 27.8%) utilized formats such as oral presentations to stakeholders and planning committees or written reports. Four recent studies published from 2016–2022 also reported utilizing cloud-based interfaces such as Forio, Dropbox or Runthemodel.com to disseminate the model, with one study uploading the model onto a web-based, user-friendly interface that allowed stakeholders to easily experiment with the model parameters online and generate corresponding charts and graphs.

## Discussion

To our knowledge, this is the first comprehensive review of public health or health care applications of GMB using well established systems science frameworks to assess the studies. Our review found that the application of GMB in public health and health care settings has increased steadily in recent years, with more than half of the papers published from 2017 to 2022. The application of GMB was most prevalent in the domains of nutrition, health care and hospital administration, and environmental health. In particular, our findings showed relatively robust work in obesity research, health care service planning, and air and water quality research. Vensim® was most commonly referenced as the software of choice, which may be attributed to its policy of making available a free version to the public and its inclusion of a feature that facilitates quick and easy causal loop diagramming, which is a core activity in most GMB projects.

Of the 72 papers in this review, 23 (31.9%) reported that the resultant models created via GMB had an eventual influence on the design and implementation of policies and programs, resulting in revisions of local action plans and strategic planning, development of new partnerships, and activation of policy leverage points [75]. The studies we reviewed revealed how SD models developed through GMB asserted their impact on policy through various mechanisms, such as by challenging pre-conceived notions of policy-makers and protecting against overconfidence by exposing potential weaknesses and unintended consequences in the system [96]. For example, a GMB session in Fiji involving officials from two government ministries led to a shared endorsement of a data sharing policy when the discussion uncovered that lack of access to evidence was a cause of poor food-related policymaking (Table 4). Another study reported how the identification of clear causal relationships between their non-profit organization’s performance indicators led to a direct revision of their budget [41]. As GMB offers a process for making these causal influences explicit while allowing for a nuanced discussion of their impacts, useful insights and opportunities for organizational or community action are able to emerge.

**Table 4:** GMB case highlights.

Exploring the dynamics of food-related policymaking processes and evidence use in Fiji using systems thinking (Waqa et al., 2017)
This study used GMB to gain insights on the factors influencing the use of evidence in food-related policy-making in Fiji, where food items associated with non-communicable diseases are produced in cheap and abundant amounts. Eighteen middle and senior managers from the country’s Ministry of Health and Medical Services (MoHMS) and Ministry of Agriculture (MOA) participated in three GMB sessions where they engaged in activities such as variable elicitation, graphs over time, connection circles, CLDs, and action ideas.
The CLD generated through the sessions revealed the following barriers to the use of evidence in food-related policy making: consultation issues, lack of engagement with stakeholders, and delays due to politics vested interests. Recognizing their unique access to the food sector, the MOA resolved to influence the consultation loop by publishing guidelines on consultation with partners. The MoHMS sought to address the issues surrounding delays by publishing strategic health communication messages to counteract the food industry’s influence on consumers choices. Both ministries agreed to endorse a government policy for data sharing to improve access to evidence and strengthen engagement among stakeholders. A guide to integrate multi-sectoral consultation and stakeholder engagement in developing policies is also currently being developed by the organizations.
Because the participants were senior staff who had organizational expertise in the policymaking process, they were able to successfully influence change within the government system. More importantly, their participation in GMB facilitated a sense of ownership over the action plans and increased coordination between the two separate ministries.
Community needs assessment among Latino families in an urban public housing development (Escobedo et al., 2019)
The Partners for Strong Healthy Families (PSHF) is a community-campus partnership that aims to improve the health and well-being of Latino families living within and around a public housing community in Los Angeles. In 2018, the organization initiated a three-hour GMB workshop with resident parents and youth to gain insight on issues surrounding parent-youth relationships and design more effective interventions to improve family wellbeing.
The graphs over time created through the GMB session highlighted differences in perspectives. The youth identified increasing household responsibilities and police presence as priority issues while the parents focused on declining respect and differences in spoken languages. Creating the CLDs allowed both perspectives to be reconciled as the participants worked together to identify the interrelationships between the different variables.
The insights gained from the GMB session had a direct impact on intervention development. The funded grant had originally planned to focus on substance use and sexual risk behaviors; however, as those weren’t identified as concerns by both parents or youth during the GMB, the intervention shifted to a bilingual, family-based meditation intervention that would improve trust, respect, and communication between families. As negative police interaction with men in the community was also characterized during the GMB, a separate intervention program to address the impact of policing tactics on the health and wellbeing of male community members was also designed. Evaluation of these programs are planned in the future to determine their effectiveness in building family and community strengths.

In addition to *insight* which was identified as an outcome in all the studies, *consensus* and *communication* were the most reported outcomes of GMB. A unique aspect of GMB is its flexibility in accommodating multiple worldviews and experiences from a wide range of stakeholders. The studies in this review described how GMB created an environment for diverse participants to express individual experiences and then build a collective model and action plan to address the shared problem. While disagreements regarding the relationships between variables were common in the initial phases of the process as participants made their mental models explicit, the participatory nature of the method allowed for productive conversations that eventually negotiated and reconciled viewpoints [36, 48, 60]. For example, a graphs-over-time exercise in one GMB session revealed differing answers among parents and youth regarding factors that affect familial relationships [37]. However, the subsequent CLD exercise allowed the two groups to bring those different variables together and explore how they interact in the system, resulting in the development of an intervention that addressed both groups’ concerns (Table 4).

Our study also underlines how GMB can be applied as a strategy for community participation and engagement [97]. Not only does the collaborative process foster a greater sense of ownership over the findings, GMB scripts such as Dots and Graphs Over Time [45, 49] also offer a unique method of eliciting information from stakeholders that traditional approaches such as interviews or surveys may not be able to draw out. Further, the outputs created such as graphs, CLDs, and simulation models can be used as springboards for future engagement with the community, with one study mentioning a plan to follow-up their GMB sessions with a community co-design workshop to develop an intervention for the identified issues [98].

Our review also showed that *systems change* (n = 14, 19.4%), *behavior change* (n = 11, 15.3%), and *positive results of system change* (n = 9, 12.5%), were the least observed outcomes of GMB. As these three outcomes usually take time and may require considerable resources to realize, they may have just not yet been evident in the rest of the studies at the time of publication. This has been documented as a continuing challenge in the community-based SD field, as the delay between participation in GMB and the development of institutional initiatives has been found to reach up to several years or more [3]. Indeed, the papers in this review that reported the positive occurrence of these outcomes were those that had considerable project duration and follow-up periods of up to five years, supporting suggestions in the field to consider the use of longitudinal designs when evaluating participatory modeling activities.

Given our findings, our review has identified several recommendations for future applications of GMB in public health and health care settings. First, transdisciplinary stakeholder involvement is a key contributor to success [36, 56, 76, 99]. A closer evaluation of the 23 papers that reported an influence on implementation revealed that bringing in multiple perspectives was crucial to creating rich discussions and achieving acceptance of the model, with one study noting that diverse stakeholder participation led to lower rates of model rejection [99]. Studies that were unable to schedule single sessions with their desired stakeholders maximized participation by implementing strategies such as creating multiple expert panel sessions or conducting lunch-and-learn sessions during convenient times [79, 100]. It should be noted that in these papers, program implementers (n = 14, 60.9%), coalition members and advocacy groups (n = 11, 47.8%), and government officials (n = 10, 43.5%) were the most widely represented stakeholder groups in the sessions.

Second, while 43 of the 72 papers (59.7%) were published in open-source journals, only a third reported dissemination of the findings back to the stakeholders and the community through other means. As with any method involving the community and the practice of translational, participatory research, the dissemination of findings beyond scientific publication should be a fundamental activity, as journal audiences do not usually include non-academic stakeholders and the general public [101]. Dissemination of GMB findings to participants will provide a tangible deliverable, facilitate decision-making, and sustain implementation impact [36]. In addition to more traditional means of dissemination such as presentations to stakeholders and distribution of reports [36, 56, 85], the more recent published literature showed an increasing trend towards cloud-based model publication [52, 102]. Using cloud-based software expands a model’s reach, as it will allow the public, not just the modeling team, to interact with the product and run simulations from anywhere. Other software such as Stella Architect’s ISEE Exchange also allows modelers to present the structure and behavior of their models in a piece-by-piece, sequential manner, making the narrative more understandable and interesting even to those with limited modeling background.

Third, there is both a gap and inconsistency in how outcomes of GMB are reported in the public health literature. Reporting is focused on the developed CLDs, models, and simulations, but other effectiveness outcomes such as those proposed by Rouwette et al. [16] are not explicitly or consistently documented. Furthermore, only 11 (15.3%) papers indicated that they had conducted process evaluations of the sessions. More rigorous and standardized reporting on the characteristics and effectiveness of GMB are needed to advance the field scientifically and refine its application in public health and health care. For this, we propose using the guidelines and scoresheet created by Rouwette et al., which outlines the recommended context, mechanism, and outcome variables that can be used as standard measurements for assessing GMB activities [16].

Fourth, previous studies have pointed out GMB as a resource-intensive approach that requires extensive technical knowledge, suggesting that its application in public health might be restricted in settings with limited access to modeling tools or training [14, 103]. However, our review of GMB applications across multiple contexts illustrates how the methodology can be tailored to varying resource constraints and participant educational levels. For instance, a participatory modeling exercise in Zambia with small-scale farmers demonstrated how GMB could be adapted to participants with low or no formal educational background and in settings without computers or electricity. The workshop used white boards and images of harvested maize and coins to illustrate variables in the model. Regular drinking glasses filled with water were used to illustrate stock and flow and feedback loops. Evaluations of these sessions a year later showed that participants found these tools to be salient in allowing them to see the interconnected nature of the system and its behavior [61]. Given the growing expansion of GMB across various settings, more research is needed on how the methodology can further be adapted for different population groups and for low resource environments. We recommend that modelers and facilitators in these settings take on an implementation science lens and formally document and evaluate any adaptations in their approach.

Finally, system dynamics practitioners have documented that the reliability, validity and the utility of system dynamics models are associated with the quality of stakeholder engagement, which is, in turn, related to the quality of GMB session facilitation [9, 104]. Although our review describes outcomes of GMB, further evaluation on fostering and sustaining high quality participation among an appropriate mix of stakeholders is warranted. Additionally, evaluation that examines how GMB can establish and strengthen multi-stakeholder research teams, working groups, and/or coalitions should be conducted.

Our study has several limitations. First, our search may not have captured the whole breadth of the public health literature on GMB as GMB may not be properly coded as a keyword in all publications. We mitigated this by consulting seven databases and checking the reference lists of eligible articles to identify any missing studies. We also utilized different variations of GMB terminology in our keywords and tested them across the different databases to ensure that we were capturing core articles in our search. Second, our findings were dependent on the availability of the data reported in the papers. As our selected outcomes were not consistently reported across all papers, it is possible that some outcome characteristics had occurred in real life but were not documented in these publications. Finally, the application of GMB in public health may be underreported in the literature, as some GMB activities may not have been documented, especially those that were conducted by public health practitioners rather than researchers.

## Conclusion

GMB is used in system dynamics projects to convene diverse stakeholders in a facilitated, deliberative scientific process that aims to specify, test and validate models that aim to further understanding of, and action to address, complex systems problems [1, 2, 11–13]. This paper demonstrates the growing breadth of GMB activities in public health and health care, with an emphasis on informing interventions for complex health challenges and enhancing the implementation of such interventions. Our findings suggest that effective GMB is associated with higher impact models as well as tangible benefits to participants, including opportunities to learn and apply new systems thinking. While the quality of the studies varies, reflecting a longstanding need to provide more training and capacity building in this method, it is evident that GMB will become increasingly more prevalent and potent as a tool for facilitating stakeholder involvement, advancing implementation science, and ultimately, developing systems solutions in the future.

## Supporting information

S1 Table PRISMA 2020 checklist. (DOCX)

S1 File Systematic review dataset. (XLSX)
